# Annotation and extraction of age and temporally-related events from clinical histories

**DOI:** 10.1186/s12911-020-01333-5

**Published:** 2020-12-30

**Authors:** Judy Hong, Anahita Davoudi, Shun Yu, Danielle L. Mowery

**Affiliations:** 1 Department of Life Sciences and Management, University of Pennsylvania, 425 S. University Ave, Levin 101, Philadelphia, PA 19104 USA; 2 Department of Biostatistics, Epidemiology, and Informatics, University of Pennsylvania, 3700 Hamilton Walk, Philadelphia, PA 19104 USA; 3 Division of Hematology and Oncology, Hospital of the University of Pennsylvania, 3400 Spruce Street, Philadelphia, PA 19104 USA; 4 Institute for Biomedical Informatics, University of Pennsylvania, 3700 Hamilton Walk, Philadelphia, PA 19104 USA

**Keywords:** Natural language processing, Medical informatics, Temporality

## Abstract

**Background:**

Age and time information stored within the histories of clinical notes can provide valuable insights for assessing a patient’s disease risk, understanding disease progression, and studying therapeutic outcomes. However, details of age and temporally-specified clinical events are not well captured, consistently codified, and readily available to research databases for study.

**Methods:**

We expanded upon existing annotation schemes to capture additional age and temporal information, conducted an annotation study to validate our expanded schema, and developed a prototypical, rule-based Named Entity Recognizer to extract our novel clinical named entities (NE). The annotation study was conducted on 138 discharge summaries from the pre-annotated 2014 ShARe/CLEF eHealth Challenge corpus. In addition to existing NE classes (TIMEX3, SUBJECT_CLASS, DISEASE_DISORDER), our schema proposes 3 additional NEs (AGE, PROCEDURE, OTHER_EVENTS). We also propose new attributes, e.g., “degree_relation” which captures the degree of biological relation for subjects annotated under SUBJECT_CLASS. As a proof of concept, we applied the schema to 49 H&P notes to encode pertinent history information for a lung cancer cohort study.

**Results:**

An abundance of information was captured under the new OTHER_EVENTS, PROCEDURE and AGE classes, with 23%, 10% and 8% of all annotated NEs belonging to the above classes, respectively. We observed high inter-annotator agreement of >80% for AGE and TIMEX3; the automated NLP system achieved F1 scores of 86% (AGE) and 86% (TIMEX3). Age and temporally-specified mentions within past medical, family, surgical, and social histories were common in our lung cancer data set; annotation is ongoing to support this translational research study.

**Conclusions:**

Our annotation schema and NLP system can encode historical events from clinical notes to support clinical and translational research studies.

## Background

In medicine, clinical histories contained within the electronic health record (EHR) document pertinent age and temporal information that could be useful for determining a patient’s disease risk, understanding the course of a disease phenotype, and predicting patient health outcomes [1–3]. Studies suggest that patients have elevated cancer risk, if one or more family members have cancer, if these cancers occur significantly earlier in life than those with sporadic cancer in the general population, or if the patient has a personal history of other prior cancers [1, 4]. Specifically, patient clinical histories play an important role in explaining risk of developing lung cancer. Studies suggest that about 8% of lung cancers are inherited or occur as a result of a genetic predisposition [2, 5, 6]. Patients have increased risk of lung cancer when multiple family members are affected with lung cancer, particularly first-degree relatives with early-onset lung cancer [7, 8]. Smokers have as much as a 15 to 30-fold increased risk of developing cancer, particularly lung cancer, when compared with their non-smoker counterparts [9]. Occupational exposures i.e., production, manufacturing, and factory workers as well as environmental exposures i.e., air pollution when considered independently from tobacco smoking are among the top 10 causes of lung cancer mortality in the United States [10]. Therefore, better characterization of lung cancer risk may lead to improved and better targeted screening efforts, which can potentially save patient lives because earlier detection of lung cancer is known to improve survival [11].

With the rapid adoption of EHR systems with coded data collection modules e.g., family and social history modules [4], clinical histories are increasingly available in electronic, structured formats allowing for large-scale retrospective research. However, the details of age and temporally-specified clinical events—past diagnoses, risk factors, surgical interventions—for both patients and family members are not well captured, consistently codified, nor readily available to research databases for oncology studies and to clinical decision support systems for cancer risk screening. Such events are often documented in unstructured form through clinical texts i.e., discharge summaries and history and physical (H&P) notes. To allow the analysis of such data, natural language processing (NLP) technologies are becoming increasingly important [12]. Our long-term goal is to construct patient phenotype profiles of relevant clinical events from all pertinent EHR data to support a variety of clinical and translational research studies and applications. For example, determining associations between clinical histories (past medical, family, social, and occupational histories) and genetic biomarkers with lung cancer outcomes e.g., progression and mortality [13]. As a means to this end, our short-term goal is to complete the development and validation of: (1) an annotation schema that explicitly describes age and temporal information in a computable format, (2) an annotation study that demonstrates this information can be manually and reliably encoded according to the annotation schema, (3) a prototypic NLP system that demonstrates such information can be automatically, accurately, and efficiently extracted, and (4) as proof of concept, an annotation study of H&P notes that demonstrates the portability and usability of this schema for encoding pertinent historical findings for a translational research study of a lung cancer cohort.

### Annotation of age and temporal information

In the last 10 years, several clinical corpora have been created, providing temporal and semantic representations and annotations for developing NLP systems. Most annotation schemas build on top of the TimeML standard [14], which captures explicit temporal expressions such as times, dates, and durations. Elhadad et al. utilized TimeML to annotate TIMEX3 data elements for the ShARe corpus, which consists of de-identified, clinical free-text notes from the MIMIC II database [15]. Similarly, Styler IV et al. [16] annotated the “Temporal Histories of Your Medical Events” (THYME) corpus using an extension of the ISO-TimeML standard [16]. The inter-annotator agreement (IAA)^1^ achieved for this schema on the THYME corpus is 80% for events and 80% for temporal expressions.^2^ Viani et al. [17] expanded TimeML to create the CALEX schema, which captures age as a Named Entity (NE), although without attributes qualifying properties about the age mention. The IAA achieved for this schema on mental health records from the Clinical Record Interactive Search (CRIS) database was 77% overall for temporal expressions.^3^

Although there has been significant work in temporal modeling, existing annotation standards do not encode age information and have limited coverage of subjects other than the patient. These standards do not encode implicit mentions nor the degree of biological relation between the patient and other subjects important for clinical and translational research studies. Furthermore, most standards focus on annotating diseases and disorders, but other clinical events i.e., procedures and social determinants of health (SDOH), can also be relevant to patient outcomes; e.g., occupation and environmental exposures with relationships to lung cancer.

### Extraction of age and temporal information

Successful NLP systems have been developed to extract age and temporal information utilizing supervised machine learning algorithms, heuristics, and rule-based components. One notable effort is the 2012 i2b2 temporal relations challenge which provided the research community with a corpus of discharge summaries annotated with temporal information for the development and evaluation of temporal reasoning systems [18]. For event detection, statistical machine learning (ML) methods consistently showed superior performance. For example, Xu et al. [19] trained a conditional random field (CRF) name entity extraction, achieving a 92% overall F1 score for extracting events. For the detection of temporal expressions, ML and rule-based methods performed equally well, though the best systems adopted a rule-based approach for value normalization. For example, Tang et al. [20] utilized predefined regular expressions applied within the HeidelTime system, achieving 87% overall F1 for temporal expression extraction. Finally, Mowery et al. developed a rule-based age information extraction system for discerning age of onsets from death with the free-text comments of an EHR family health history module using the Fast Healthcare Interoperability Resource (FHIR) standard, achieving a F1-score performance of 94% onset and 94% death [4].

Several automated NLP tools already exist to extract explicit temporal expressions and named entities that describe disease disorders. However, we introduce new NEs and attributes in our annotation schema to capture previously un-annotated age and temporal information. As an initial step towards automation, we built a prototypical tool to assess the amount of work necessary to extract this information and to serve as a foundation for a future hybrid rule-based and ML extraction method for large datasets. Finally, we demonstrate that our expanded schema can capture pertinent historical findings from a sample of history and physical (H&P) notes for a lung cancer cohort study. By applying the schema to this subset of H&P notes from the University of Pennsylvania Health System, we aim to assess the portability and usability of the expanded schema for representing pertinent clinical histories for lung cancer research.

## Methods

**Fig. 1:**
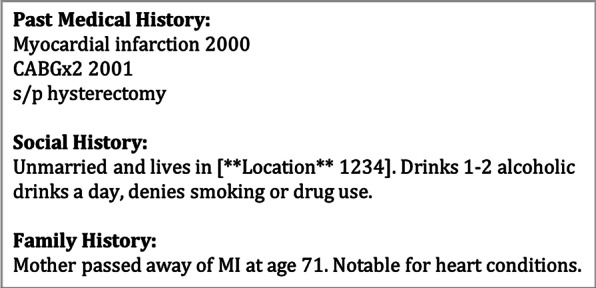
Fictitious example illustrating sections from a discharge summary, like the ShARe/CLEF dataset redacted according to Safe Harbor guidelines

In this University of Pennsylvania Institute Review Board (IRB)-approved pilot study, we leveraged the pre-annotated 2014 ShARe/CLEF eHealth Challenge corpus [21], a subset of the Medical Information Mart for Intensive Care (MIMIC)-II database [22] collected from the intensive care units of Beth Israel Deaconess Medical Center. We sampled 138 de-identified, free-text discharge summaries. To focus the search for historical rather than acute events, we extracted sections (*past medical history*, *past surgical history*, *family history*, *social history*) which have a higher likelihood of containing age and temporally-specified clinical events (Fig. 1).

### Development of annotation schema

We aimed to develop a schema that integrates Named Entities (NEs), attributes, and relationships relevant for representation of age and temporal information. To align our annotated classes with current and well-adopted annotation efforts in the NLP community, we added new and expanded existing annotation classes to the ShARe [21], TimeML [14], and CALEX [17] schemas. Additionally, new classes were constructed based on a linguistic study of 20 randomly-selected discharge summaries from ShARe corpus. Documents were annotated according to the proposed schema in batches of 5 by authors, JH, a data scientist, and DM, a clinical informaticist. After each batch, we reached consensus, updated the annotation schema, and modified the annotation guidelines. At the end of the schema development process, previously annotated documents were revised to create a final reference standard. The schema addresses information important for interpreting a patient’s clinical history: (1) NEs, (2) attributes and their values, and (3) relationships between NEs.

### Named entities, attributes, and relationships

The existing annotation scheme for the 2014 ShARe/CLEF eHealth Challenge corpus included NE classes: TIMEX3 (T), DISEASE_DISORDER (DD), SUBJECT_CLASS (S). We propose 3 new NEs: AGE (A), PROCEDURE (P), OTHER_EVENTS (OE) due to their relevance to clinical and translational research studies. We also describe a new attribute type, degree_relation, for the pre-existing SUBJECT_CLASS (S) and expand the class to include implicit mentions.

For each NE below, we define boundaries—start and end offsets—for the NE span in the text with square brackets followed by a subscript indicating the annotation type e.g., [left knee arthroscopy]_P_ is a spanned PROCEDURE mention.

- TIMEX3 (T): describes any text span that specifies a temporal expression about a clinically-relevant event (i.e. DISEASE_DISORDER, PROCEDURE, or OTHER_EVENT). TIMEX3 has attribute types: date, time, and duration.
  Ex. “Arthroscopy in [1997]_T_”, Type: date.
- AGE (A): describes any text span that specifies a subject’s age or the age at which a clinically relevant event occurred. AGE has the attribute types: fully-specified, less-specified, event-specified. It can be normalized to an age range (e.g. START 70, END 79) and time range (e.g. START 10/10/1950, END 10/10/1959).
  Ex. “Patient had [childhood]_A_ diabetes”, Type: event-specified.
- SUBJECT_CLASS (S): describes any span of text that refers to a subject that is not the patient. While the ShARe schema only annotates explicitly-mentioned entities experiencing a DISEASE_DISORDER, we expand SUBJECT_CLASS to include any subject that is not the patient. We also include implicit references to other subjects, commonly found within the *family history* sections. Ex: Subject explicitly referenced: “[father]_S_ had [CAD]_DD_”. Subject implicitly referenced: “[family]_S_ history: notable for [CAD]_DD_”. Following from the ShARe schema, SUBJECT_CLASS can be normalized to: family_member, donor_other, donor_family_member, other.
  A novel attribute type in our schema describes the subject’s degree of biological relation, degree_relation: 0, 1, 2, 3, not_biologically_related, unknown. In clinical and translational research studies, capturing the degree of relation is important for studying and determining disease heritability within families [1]. Degree of relation is based on genetic similarity to the patient [23] e.g., 0th = identical twin, 1st = parent, siblings, offspring.
  Ex. “[Sister]_S_ had breast cancer”, Degree_relation: 1.
- DISEASE_DISORDER (DD): describes any span of text that can be mapped to a concept in the Systematized Nomenclature of Medicine-Clinical Terms (SNOMED-CT) terminology, which belongs to the Disorder semantic group. DISEASE_DISORDER has the attribute DocTimeRel: after, overlap, before_overlap, before, and unknown which specifies the temporal relation between the entity and the time of document creation. The DocTimeRel attribute is critically important for encoding the relative time of a clinical event when more explicit and informative temporal expressions are not provided in the text. The entity has the attribute associatedCode, which specifies the Unified Medical Language System (UMLS) Concept Unique Identifiers (CUI) that best describes the entity.
  Ex. “Patient with [end-stage renal disease]_DD_”, DocTimeRel: before_overlap, associatedCode: C2316810: chronic kidney disease stage 5.
- PROCEDURE (P): describes any span of text that can be mapped to a concept in the SNOMED-CT terminology, which belongs to the Procedure semantic group. Similar to DISEASE_DISORDER, PROCEDURE has the attribute DocTimeRel: after, overlap, before_overlap, before, and unknown which specifies the temporal relation between the entity and the document creation time. PROCEDURE also has the attribute associatedCode, which specifies the UMLS CUI that best describes the entity.
  Ex. “[appendectomy]_P_ scheduled for next week”, DocTimeRel: after, associatedCode: C0003611: appendectomy.
- OTHER_EVENTS (OE): describes any social determinant of health (SDOH) or other events that could be clinically relevant. OTHER_EVENTS has attribute type with values: martial status, death, good health, substance use, occupation, exposure, living situation, outcome of procedures, and other. This novel entity will help us understand how existing annotation standards can be expanded to include more clinically relevant events. OTHER_EVENTS also has the attribute DocTimeRel and the optional attribute associatedCode, which specifies the UMLS CUI for mentions of substance use only.
  Ex. “Patient is a [factory worker]_OE_”, DocTimeRel: before_overlap, type: occupation.

### Annotation study

Each document was pre-annotated with certain NEs and attributes (TIMEX3, DISEASE_DISORDER, SUBJECT_CLASS) from the 2014 ShARe/CLEF eHealth Challenge. The aim of this annotation task is to expand these existing annotations according to our more detailed annotation schema. Specifically, annotators were instructed to do the following:

1. Annotate new NEs (AGE, PROCEDURE, and OTHER_EVENTS)
2. Identify new spans of text under the expanded definition of certain NEs (e.g. annotating “wife” as a relevant SUBJECT_CLASS)
3. Add new attributes for existing NEs (degree_relation for SUBJECT_CLASS)
4. Link NEs with relationships when multiple NEs are required to fully capture the description and semantic meaning of a clinical event, e.g. what was the type of clinical event, who experienced it, when it was experienced.

In the sentence, “[Father]_S_ [died]_OE_ of [MI]_DD_ at [age 69]_A_.”, the annotated relationships include:

- OTHER_EVENT (OE)-to-SUBJECT_CLASS (S)
- DISEASE_DISORDER (DD)-to-SUBJECT_CLASS (S)
- OTHER_EVENT (OE)-to-AGE (A)

Annotations were carried out by DM and AD from the Semantic Analysis of Text to Inform Clinical Action (SemAnTICA) laboratory of the University of Pennsylvania using the extensible Human Oracle Suite of Tools (eHOST) annotation tool [24]. Over the course of one week, JH trained both annotators with the annotation schema and reviewed how to apply the schema to clinical notes leveraging the annotation software. To reduce the likelihood of annotator fatigue due to the schema’s complexity, we assigned the majority attribute value for the previously annotated conditions as default values. Annotators were instructed to change default values to semantically represent the mention in the text. Annotators were trained with batches of 5 documents each, and annotator performance inter-annotator agreement (IAA) was measured using the F1-score, the harmonic mean of recall and precision. The F1-score was calculated between each annotator and the reference standard using the eHOST built-in IAA report generator. Annotation performance was assessed using three levels of agreement determination: (1) NE, (2) NE + attributes, (3) NE + attributes + relationships.

Then, the annotators were given two weeks to independently annotate mutually-exclusive note sets each (n = 59 discharge summaries), to produce an annotated corpus of 138 documents in total (inclusive of the development set of 20 discharge summaries).

To assess the utility of novel elements in our annotation schema, we report the distribution of annotated classes and attributes. We also report the distribution of other events including martial status, death, good health, substance use, occupation, exposure, living situation, outcome of procedures, and other mentions observed in our corpus.

The resulting annotations were leveraged to develop and evaluate an NLP pipeline for the automated extraction of these semantic and temporal classes as a traditional named entity recognition (NER) task.

### Automated named entity recognition

**Fig. 2:**
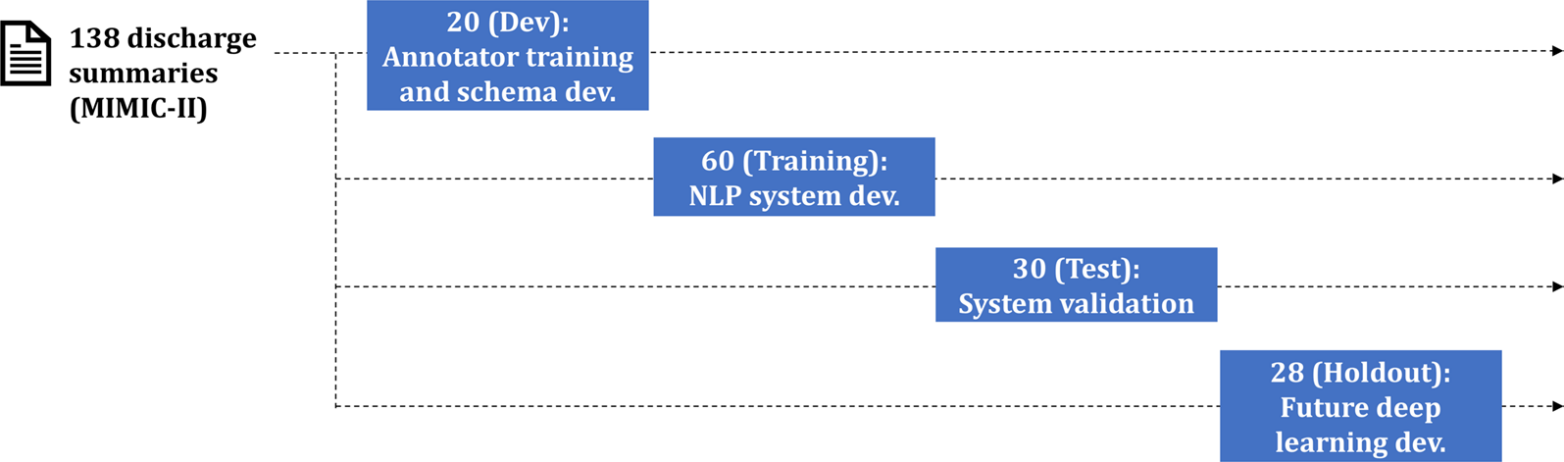
Workflow of annotation study

The annotated corpus was randomly split into development, training, test, and future holdout sets in a 15:45:20:20 ratio, respectively. The development set facilitated annotator training and schema development. The training set was used for manual rule engineering and NLP system development, with validation on the test set. The holdout set was set aside to validate deep learning components in future work. This workflow is illustrated in Fig. 2.

Leveraging the training set, we developed two NLP modules to extract NEs from the clinical texts. The first module extracted the AGE, SUBJECT_CLASS and TIMEX3 entities using rule-based matching; the second module extracted the DISEASE_DISORDER and PROCEDURE entities using QuickUMLS [25]. Both modules were built and integrated using spaCy V2.1, an open-source software library for advanced NLP. Integration within a spaCy pipeline supports future integration with deep learning packages and fast information extraction leveraging a Cython compiler.

In the first module, we extracted non-medical NEs: AGE, SUBJECT_CLASS, and TIMEX3 using a rule-based system. This system relies on a number of features, namely the section in which the entity is found and the pattern of its text mention. More specifically, section labels were identified using a set of keywords (e.g. “past medical history”, “family history”). We trained spaCy’s EntityRuler to extract new NEs based on pattern dictionaries and regular expressions. For example, AGE can be identified with patterns combined with regular expressions: “##-year-old, ## yo”, where # is a numeric. The trained EntityRuler was added to the spaCy pipeline using nlp.add_pipe.

In the second module, we extracted medical NEs: DISEASE_DISORDER and PROCEDURE using QuickUMLS against the UMLS Metathesaurus [26].^4^ QuickUMLS is a fast, unsupervised, approximate dictionary-matching algorithm for medical concept extraction [25]. Compared to other state-of-the-art entity extraction tools including MetaMap and cTAKES, QuickUMLS achieves similar precision and recall, but is 135 times faster; thus, scalable to large datasets. The QuickUMLS module receives notes as input and returned a set of spans in the notes as well as UMLS concepts associated with each span. We integrated the output from this module as a post-processing step after applying spaCy’s EntityRuler. The training set was used to tune QuickUMLS to extract any concepts from the UMLS semantic types within Table 1. Notably, the inclusion or exclusion of semantic types resulted in trade-offs between precision and recall (to be discussed further in the “Discussion” section).

**Table 1:** Included UMLS semantic types for entity extraction

NE	UMLS semantic types
DISEASE_DISORDER	T047: Disease or Syndrome
	T048: Mental or behavioral dysfunction
	T046: Pathologic function
	T191: Neoplastic process
PROCEDURE	T062: Therapeutic or Preventative Procedure

**Fig. 3:**
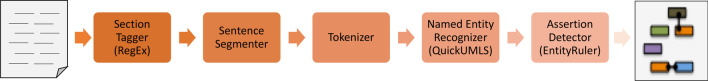
NLP pipeline components

For this study, attribute normalization and relation detection were outside of scope and will be left to future work. We integrated the EntityRuler and QuickUMLS modules as the final components of the spaCy NLP pipeline, which consists of section tagger, sentence segmentor, tokenizer, named entity recognizer, and assertion detector modules (Fig. 3).

### Evaluation

We evaluated the performance of the automated NER pipeline on the validation set of 30 notes, using a customized python (v3.7.0) script. Specifically, we defined matches between the NER pipeline extractions and the reference standard annotations using overlapping spans. For example, a true positive was defined as overlapping annotations assigned to the same NE type. We counted the number of true positives (TP: system’s span occurs in the annotated corpus), false positives (FP: system’s span does not occur in the annotated corpus), and false negatives (FN: system did not identify a span in the annotated corpus). We computed recall to determine the proportion of the annotated corpus spans that the system identified, and precision to determine the proportion of correctly-identified spans by the system. We also measured the F1-score (i.e., the harmonic mean of precision and recall) to quantify overall agreement for the NER tasks [27].

### Lung cancer demonstration study

As part of an ongoing translational research study, we aim to determine associations between clinical histories (past medical, family, and social histories) and genetic biomarkers with lung cancer outcomes e.g., progression and mortality. To demonstrate that the schema can represent and capture pertinent historical information for determining age and dates of past and current cancer diagnoses, pertinent lung cancer diagnostic procedures and therapies, familial cancers, and social histories/exposures, authors SY, a clinical oncologist and DM, applied the schema with consensus review to 49 history and physical (H&P) notes from the University of Pennsylvania Health System for patients with confirmed stage IIIB+ non-small cell lung cancer. We report initial descriptive statistics for historical events with age/time specifications and degree of relation for family histories when applicable.

## Results

### Annotator training

**Fig. 4:**
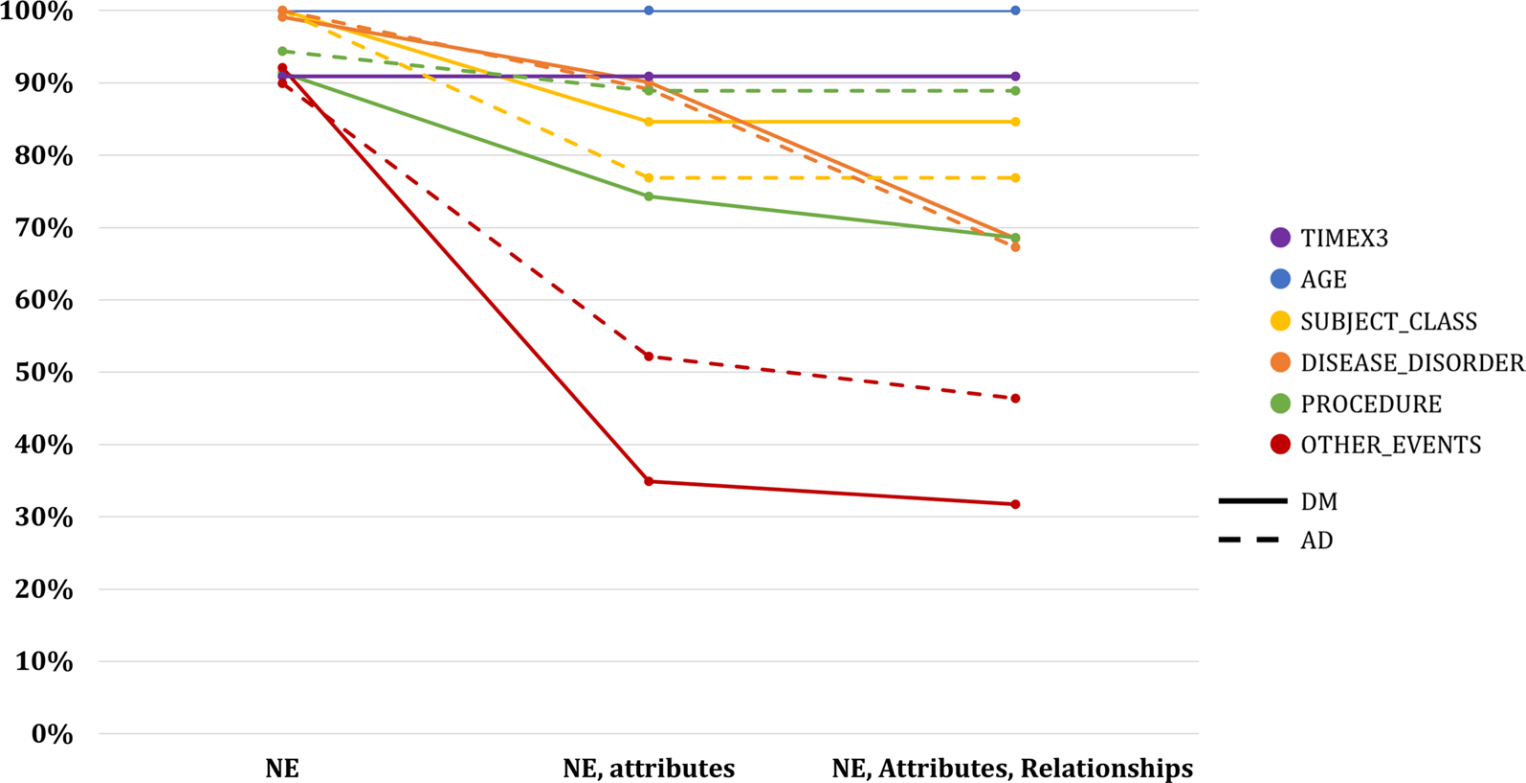
IAA between annotator and reference (Batch 3 and 4)

We report inter-annotator agreement with the initial reference standard (n = 20 documents) according to the three levels of match determination for NEs, their attributes, and relationships between them in Fig. 4.

Annotator agreement generally decreases as match criteria become stricter. Notably, agreement for the AGE and TIMEX3 classes remain unchanged even after attributes and relationships are added. This indicates that the annotation schema is well-designed for these classes and/or that these classes are easier to annotate. For other classes, a noticeable drop in agreement occurs when attributes are included.

For the annotation of NEs only, annotators achieved nearly 100% agreement with the reference standard across all classes. When the annotation of attributes and relationships is included, annotators achieved high agreement (>91%) for the AGE, TIMEX3, and SUBJECT_CLASS, but agreement was poorer (<70%) for DISEASE_DISORDER, PROCEDURE and OTHER_EVENTS. For DISEASE_DISORDER and PROCEDURE, this is largely attributable to differing allocation of UMLS CUIs. For OTHER_EVENTS, which captures relevant SDOH, more training is required to achieve a consistent allocation of attribute values.

We compared our performance to the widely used ISO-TIMEML standard. When applied on the THYME corpus, the standard achieved IAA of 96%^5^ for NE annotation, and 80% and 80% for attribute annotation for events^6^ and TIMEX3, respectively [16]. Our schema achieves similar or better annotator agreement for NE annotation, but lags in agreement for attribute annotation. In future work, we aim to expand annotator training and further refine the annotation schema to promote consistency between annotators. For the purpose of developing an automated NER extraction system, annotators need to annotate NEs consistently. Based on the IAA achieved at the end of the training period for annotation of NEs, annotators were able to proceed with single-annotation of the full dataset.

### Annotation study

**Fig. 5:**
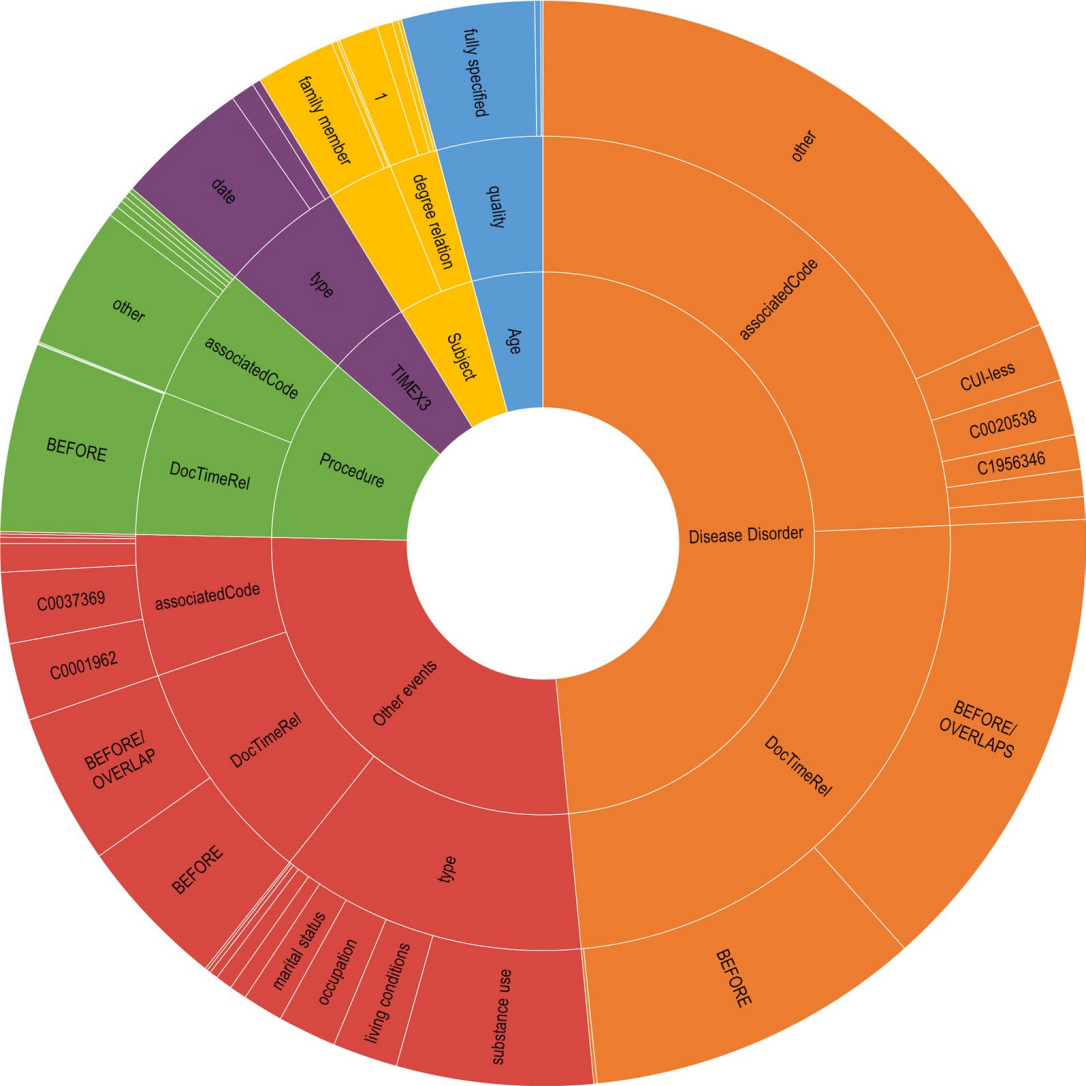
Distribution of class values

We report the distribution of NEs in the full corpus of 138 discharge summaries in Table 2 and Fig. 5. Only NEs in the specific sections (past medical history, past surgical history, family history, social history) are annotated. Excluding the holdout set, the full corpus has a total of 1540 NEs with a distribution of: 688 (45%) DISEASE_DISORDERs, 358 (23%) OTHER_EVENTS, 161 (10%) PROCEDURE, 140 (9%) TIMEX3, 119 (8%) AGE, and 74 (5%) SUBJECT_CLASS. Distribution of NEs within development, validation, and testing are similar.

**Table 2:** Distribution of NEs from the full corpus

	Development	Train	Test	All (excl. holdout)
# of documents	20	60	30	110
All	306 (100%)	786 (100%)	448 (100%)	1540 (100%)
TIMEX3	28 (10%)	76 (10%)	36 (8%)	140 (9%)
AGE	23 (8%)	60 (7%)	36 (8%)	119 (8%)
SUBJECT	18 (6%)	32 (4%)	24 (5%)	74(5%)
DISEASE_DISORDER	127 (45%)	373 (49%)	188 (42%)	688 (45%)
PROCEDURE	37 (12%)	71 (9%)	53 (12%)	161 (10%)
OTHER_EVENTS	73 (18%)	174 (21%)	111 (25%)	358 (23%)

The majority of NEs are DISEASE_DISORDERS and OTHER_EVENTS. Though not of the highest proportions, there is an abundance of age and time-related data, with an average of 1.27 mentions per document for TIMEX and 1.08 mentions per document for AGE.

In Table 3, we report the distribution of social determinants of health from the training set of 60 documents. The most frequent SDOH event mention was substance usage (including tobacco, illicit drugs, and alcohol) (56%) followed by living situation (17%) and occupation (13%).

**Table 3:** Distribution of social determinants of health (SDOH) with examples from the training corpus

SDOH	Examples	Counts
Substance use	Ex-smoker, drinks 1 bottle of vodka/day	87 (56%)
Living situation	Lives in a nursing home	26 (17%)
Occupation	Retired, nurse	20 (13%)
Martial status	Married, widow	8 (5%)
Death	Passed away, died	6 (4%)
Exposure	Exposed to asbestos	3 (2%)
Other	Does not speak English	5 (3%)

### Automated named entity recognition

**Fig. 6:**
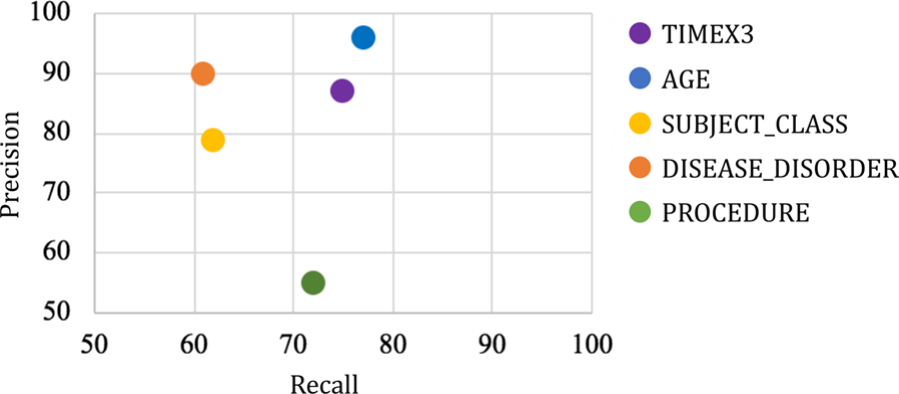
Results of automated NER system

From the testing set (n = 30 documents), the results of the automated NER system are shown in Fig. 6. Overall classes, recall was moderate (65%) and precision was high (83%), demonstrating promising results. Less prevalent cases of TIMEX, AGE, and PROCEDURE were extracted with higher recall than other more prevalent classes. The highest precision performance was achieved for TIMEX, AGE, and DISEASE_DISORDERS.

### Lung cancer demonstration study

From the annotated set of 49 sampled history and physical (H&P) notes from the University of Pennsylvania Health System (Table 4), we observed that 47% of past medical history mentions, 65% of surgical history mentions, and less than 1% of family history mentions were age or temporally-specified using dates. Most mentions of family history describe other cancers (89%) rather than lung cancer diagnoses (11%). Using the DocTimeRel attribute, most mentions of social history (92%) describe former smoker (DocTimeRel: before) and current (DocTimeRel: before_overlaps) smoker descriptions. No occupational or environmental exposure information was observed within the patient social histories.

**Table 4:** Distribution of mentions related to past medical history, past surgical history, family history, and social history for stage IIIB+ lung cancer patients from 49 sampled H&P notes

Section variable type	Variable	Examples	Counts
Past medical history			73
	Lung cancer	Stage III NSCLCA	23 (32%)
	Lung cancer with date	Neoplasm NOS-lung nodule #/#/####	12 (16%)
	Other cancer	Renal cancer; brain metastasis	16 (22%)
	Other cancer with date	Bladder cancer #/#/####	20 (27%)
	Other cancer with age	Neoplasm of prostate...age ##	2 (3%)
Surgical history			52
	Procedures	XRT, wedge resection of LUL	18 (35%)
	procedures with date	Lung bx ##/##; lobectomy ####	34 (65%)
Social history			66
	Smoking—negated (non-smoker)	Tobacco use: never	5 (7%)
	Smoking—before (former smoker)	Tobacco use: quit	48 (73%)
	Smoking—before_overlaps (current smoker)	Current everyday smoker	13 (20%)
Family history			55
	Lung cancer w/ 1st degree relative	Lung cancer in her sister	2 (4%)
	Lung cancer w/ 2nd degree relative	Lung cancer maternal uncle	2 (4%)
	Lung cancer w/ age	Lung cancer maternal uncle ##s	2 (4%)
	Other cancer w/ 1st degree relative	Ovarian cancer mother	31 (56%)
	Other cancer w/ 2nd degree relative	Breast cancer maternal aunt	15 (27%)
	Other cancer w/age	Breast cancer mother age ##s	3 (5%)

## Discussion

For this pilot study, we (1) introduced and applied an expanded annotation schema that supports the extraction of age and temporally-specified information, (2) developed a prototypic, rule-based NLP system to extract clinical events with age and temporal mentions, and (3) applied and assessed the portability and usability of the expanded schema for representing pertinent clinical histories for lung cancer research.

### Annotation of age and temporal information

In first annotation task, two annotators expanded upon 138 pre-annotated discharge summaries from the 2014 ShARe/eHealth Challenge. The annotators were instructed to add new classes, attributes and relationships, as well as to expand the definition of existing classes.

Annotator training and feedback yielded some notable observations. Firstly, AGE and TIMEX3 classes were placed in consistent locations through the text and followed easily identifiable patterns likely explaining fewer inter-annotator inconsistencies. Other classes required extensive training to achieve acceptable IAA for attribute annotation. For example, the DocTimeRel attribute (for DISEASE_DISORDER, PROCEDURE and OTHER_EVENTS) required more annotator judgement on the nature (before_overlaps = acute or chronic) or focus on the grammatical description of the event (before = a disorder that resolved in the past, e.g. “sister had breast cancer” versus before_overlaps = a disorder that continues into the present, e.g., “sister has breast cancer”). Another notable challenge is the allocation of the associatedCode attribute (for DISEASE_DISORDER, PROCEDURE and OTHER_EVENTS) which required annotators to choose a UMLS CUI that best describes the entity. Because one medical concept can be described by many UMLS CUIs (e.g. “foot surgery” can be described by both C0188413: Operative procedure on foot and C1552280: Surgery, Foot), this was a major source of inconsistency.

We hypothesized that specific sections of the discharge summaries—*past medical history*, *past surgical history*, *family history*, *social history*—have a high likelihood of containing age and temporally-specified clinical events. In the 2014 ShARe/eHealth data set, we observed an average of 1.27 TIMEX3 mentions per document and 1.08 AGE mentions per document. Mentions of subjects other than the patient occurred at an average of 0.71 mentions per document. TIMEX3 mentions indeed occurred most frequently in the *past medical history* section; they were often used to describe the DISEASE_DISORDER and PROCEDURE classes (e.g. “s/p appendectomy 2001”, “motor vehicle accident 05-13-2003”). Unsurprisingly, AGE and SUBJECT_CLASS mentions occurred most frequently in the *family history* section; they were often used to describe the health status of family members (e.g. “61yo mother has HTN”).

### Utility of novel elements in annotation schema

We proposed 3 new NEs (AGE, PROCEDURE, OTHER_EVENTS) and describe a new attribute type degree_relation for the pre-existing SUBJECT_CLASS to the 2014 ShARe/CLEF schema. We analyzed the distribution of NEs and corresponding attributes to assess their utility. We observe that a rich amount of information was captured under the new OTHER_EVENTS, PROCEDURE, and AGE classes. 23%, 10% and 8% of all annotated NEs belonged to the above classes respectively.

The social history section contain an abundance of OTHER_EVENTS that describe clinically relevant Social Determinants of Health (SDOH). Across the full 2014 ShARe/eHealth corpus, we observed that the most frequent SDOH events included substance usage. This is unsurprising given the extensive knowledge of substance usages impact on human health. We also observed a significant number of discussions about patient’s living situation e.g., living alone or with a spouse or family. These mentions provide important information about potential care providers and individuals available to provide social support. In the dataset, we were more likely to observe documented indirect exposures e.g., occupation (police officer exposed to violence or nurse exposed to infectious disease) rather than direct exposures (asbestos and other chemicals). Although infrequent in our dataset, at scale, this information can be important for calculating risk for disease. In future work, we will leverage the NIOSH Industry and Occupation Computerized Coding System (NIOCCS) for encoding information about common exposures when occupation is provided [28].

The past medical history and past surgical history sections contain an abundance of information relating to past medical procedures that were previously un-annotated under 2014 ShARe/CLEF eHealth Challenge guidelines. For example, a past medical history section as illustrated in Fig. 1 contains mentions of “myocardial infarction”, “CABG” and “hysterectomy”. “myocardial infarction” and “CABG” are explicit mentions of disorders and thus were already captured under the DISEASE_DISORDER class, but “hysterectomy” is a medical procedure and was previously un-annotated. Mentions of medical procedures are important because they can be used to imply past disease or preventive measures. For example, a patient may undergo hysterectomy due to a past history of endometriosis, heavy periods or cancers. Under the new PROCEDURE class, an average of 1.43 mentions of medical procedures were captured per document.

The new degree_relation attribute under SUBJECT_CLASS show that the majority of non-patient subjects have a first degree of relation to the patient (61%). AGE mentions are often fully-specified (94%) rather than less-specified or event-specified suggesting most ages do not require estimation to be readily useful.

### Extraction of age and temporal information

While automated NLP tools already exist for the extraction of explicit temporal expressions and disease disorders, we introduce new NEs and attributes in our proposed schema. The goal of this automated NLP component is to build a prototypical tool to (1) assess the amount of work necessary to extract such new information, and (2) serve as a foundation for a future hybrid rule-based and ML extraction method.

We conducted a systematic review of errors and omissions generated by our NLP pipeline to inform next steps. We observed the following distribution of errors: 18% missed sections, 13% semantic type disagreement, 40% acronyms/abbreviations, and 29% QuickUMLS error due to misspelling/missed term. To improve the sensitivity of the section detection, we are actively developing a more robust section tagger leveraging a hybrid approach powered by the SecTag terminology, dictionary-matching, and word embeddings. Other errors, addressed below, had more profound impact on the extraction of non-medical and medical NEs from the training corpus.

Non-medical NEs (AGE, TIMEX3 (DATE), and SUBJECT_CLASS) are generally well-recognized with rule-based techniques, as evidenced by the high recall by the automated NER system (77% for AGE, 75% for TIMEX3 (DATE), 62% for SUBJECT_CLASS). In particular, age and temporal information within discharge summaries follow regular patterns (e.g. “XX-year-old”, “YYYY-MM-DD”). Some challenges remain. Other types of numeric information (e.g. quantity of medication, location identifier) can occur in similar patterns as dates, leading to false positives for temporal information. Within the *family history* sections, references to other subjects are often implicit (e.g. “notable for CAD” instead of “father had CAD”) and difficult to extract through regular expressions and will require integration of document structure to discern the subject experiencer for these disorder mentions. While age information does conform to certain patterns, a wide range of patterns may occur and any rule-based system attempt is brittle. One opportunity to improve the recall of AGE, TIMEX3, and SUBJECT_CLASS NEs include incorporating more lexical and syntactic features to capture surrounding context and deep learning approaches to improve recall of similar mentions.

Medical NEs (DISEASE_DISORDER and PROCEDURE) are not well-recognized presenting as a challenging task. Dictionary matching using QuickUMLS against the UMLS Metathesaurus is a fast and easily implementable approach that exhibits moderate to high recall (61% for DISEASE_DISORDER, 72% for PROCEDURE). Notably, the system also exhibits high precision (90%) for DISEASE_DISORDER. One main limitation of this approach is that QuickUMLS is not sensitive to the use of short forms such as acronyms and abbreviations, contributing to only a moderate recall for DISEASE_DISORDER. Furthermore, the inclusion or exclusion of UMLS semantic types result in a precision-recall tradeoff. For example, a “prostate biopsy” is a clinically relevant procedure that the system should capture, but including the UMLS semantic type T060: Diagnostic procedure greatly lowers precision. One possible solution to improve the recall for DISEASE_DISORDER NE extraction could be to develop an integrate an acronym and abbreviation module; this module would identify, extract, and disambiguate acronyms leveraging the 2013 ShARe/CLEF eHealth Challenge dataset [29]. A potential solution to improve the precision of DISEASE_DISORDER NE extraction is to develop a post-processing model to filter out spurious semantic spans identified by QuickUMLS.

### Lung cancer demonstration study

For our ongoing translational research study, we aim to determine associations between clinical histories and genetic biomarkers with lung cancer outcomes e.g., progression and mortality [30]. We demonstrated that expanded annotation schema could capture detailed, pertinent clinical histories from H&P notes for our lung cancer cohort. By capturing times of initial diagnoses with lung cancer, we can compute time durations to study outcomes e.g., progression and mortality, for new patients transferred for care late in their cancer course. We can also determine whether all or some diagnostics and therapies were received outside the University of Pennsylvania Health System to estimate data completeness for each patient. Furthermore, by encoding degree of relation and age-specified diagnoses (personal and familial) as well as social histories, we can study the influence of familial heritability and environmental/occupational exposures with lung cancer outcomes [13].

### Future work

To further validate the utility and portability of our annotation schema, we aim to conduct annotation studies on other datasets for which capturing age, temporal and family history information have strong clinical utility.

For the extraction component, our results illustrate how rule-based systems can extract medical information with good accuracy when the to-be-extracted information follows regular patterns. A prototypic rule-based system utilizing only regex expressions achieves high precision and recall for AGE, indicating that further revision to the regular expressions and rule-based logic may be sufficient to achieve higher performance for certain NEs. However, as evidenced by the poorer performance for DISEASE_DISORDER and PROCEDURE, rule-based systems are brittle and don’t scale well to complex patterns (e.g. description of medical events) or short forms (e.g. non-standardized abbreviations). For those NEs, ML-based methods may prove beneficial. Recently, deep learning neural networks have been especially successful in complex clinical NER tasks [31]. However, deep learning methods such as neural networks remain dependent on the availability of large annotated datasets, which is a significant hurdle for electronic health records (EHRs). Namely, only a few publicly available datasets exist for medical NLP, and even fewer exist with annotations for NLP tasks. Thus, the challenge is to develop a hybrid rule-based and ML method that is applicable to small datasets. For example, pre-trained custom word embeddings [32] can be applied after dictionary matching to improve classification of clinical events. The current NLP system is built within spaCy, which allows for easy incorporation of deep learning models in the future. Finally, at the time of print, our lung cancer annotation study had just started. We will continue to annotate our lung cancer cohort for detailed history information (first manually then automatically using our NLP pipeline) to support our translational research study of the lung cancer cohort.

## Conclusion

In this pilot study, we expanded upon the ShARe Semantic Schema to support the representation of age, temporal and family history information. Specifically, we introduced a new AGE class that allows for the characterization of age information. We expanded SUBJECT_CLASS to include any subject that is not the patient, even if implicitly mentioned; we also introduced a new attribute degree_relation to the SUBJECT_CLASS, which allows for the capture of biological relation which is significant for assessing disease risk that has a genetic heritability. Lastly, we introduced the PROCEDURE class as they are commonly related to age and temporal information and can be placed on a patient timeline.

We then applied this annotation schema on the 2014 ShARe eHealth Challenge corpus, which is one of the few publicly datasets for clinical NLP. A rich amount of information was captured under the new OTHER_EVENTS, PROCEDURE, and AGE classes, illustrating the utility of the novel elements of our annotation schema. Our annotated corpus, once further validated by more annotators as a reference standard, can be released as a resource for the clinical NLP community. We foresee this corpus to be particularly valuable for researchers who conduct research in which the age and timing of clinical events is critical, e.g., disease risk and symptom onset.

We then build a prototypical NLP tool to assess the amount of work necessary to extract such new information, and to serve as a foundation for a future automation efforts. A preliminary rule-based system utilizing regular expressions for non-medical NEs and dictionary matching (QuickUMLS) for medical NEs showed promising results. Most notably, age information as captured through the AGE class was well extracted by the rule-based module of our NLP pipeline. Further work can be done to integrate deep learning models into the existing spaCy pipeline to improve extraction of medical NEs. The long-term goal is to develop a hybrid rule-based and deep learning NLP system to automatically extract age and temporal information, for the construction of longitudinal clinical profiles for any patient, including our lung cancer cohort.

## Data Availability

Data is available upon request and through MIMIC approval.

## Abbreviations

EHR: Electronic Health Record
UMLS: Unified Medical Language System
NLP: Natural Language Processing
NE: Named Entity
NER: Named Entity Recognition
ML: Machine Learning
H[MYAMP: P] History & Physical
ShARe: Shared Annotated Resources
CLEF: Conference and Labs of the Evaluation Forum
CAD: Coronary Artery Disease
CABG: Coronary Artery Bypass Graft
NIOSH: National Institute for Occupational Safety and Health
NIOCCS: NIOSH Industry & Occupation Computerized Coding System
SDOH: Social Determinants of Health
HTN: Hypertension
IAA: Inter-Annotator Agreement
CUI: Concept Unique Identifier
SemAnTICA: Semantic Analysis of Text to Inform Clinical Action
eHOST: extensible Human Oracle Suite of Tools
MIMIC: Medical Information Mart for Intensive Care
FHIR: Fast Healthcare Interoperability Resource
CRIS: Clinical Record Interactive Search
CRF: Conditional Random Fields

## Footnotes

1 F1 scores are used to represent IAA.

2 Match criteria include both span and attributes.

3 Match criteria include span only.

4 The Unified Medical Language System is a collection of medical thesauri maintained by the United States National Library of Medicine.

5 IAA as represented by F1 scores.

6 Most similar to DISEASE_DISORDER.
